# Abdominal aortic aneurysms part one: Epidemiology, presentation and preoperative considerations

**DOI:** 10.1177/1750458920954014

**Published:** 2020-09-28

**Authors:** Holly N Hellawell, Ahmed M H A M Mostafa, Harry Kyriacou, Anoop S Sumal, Jonathan R Boyle

**Affiliations:** 1 University of Cambridge, School of Clinical Medicine, Addenbrookes Hospital, Cambridge, UK; 2 Cambridge University Hospitals, NHS Foundation Trust, Cambridge Vascular Unit, Cambridge, UK

**Keywords:** Abdominal aortic aneurysm, Cardiovascular disease, Preoperative period, Vascular surgery, Screening

## Abstract

An abdominal aortic aneurysm is an irreversible dilatation of the abdominal aorta. The majority of abdominal aortic aneurysms are asymptomatic and identified incidentally while investigating a separate pathology. Others are detected by national screening programmes and some present due to a growth or rupture. Symptomatic or ruptured aneurysms require urgent or emergency repair in patients fit for surgery. Perioperative practitioners should therefore be aware of how patients with abdominal aortic aneurysms present and are investigated, so that they can implement timely management. Guidelines have been recently updated to reflect this. This literature review discusses these recommendations and explores the evidence upon which they are based. The aim of this article is to highlight the important preoperative principles that need to be considered in cases of abdominal aortic aneurysm.

**Provenance and Peer review:** Unsolicited contribution; Peer reviewed; Accepted for publication 9 August 2020.

## Introduction

An abdominal aortic aneurysm (AAA) is an irreversible dilatation of the abdominal aorta to a diameter greater than 3.0cm or 1.5 times its normal anteroposterior diameter (NICE 2020). In 2019, Public Health England (PHE) reported that approximately 0.97% of men screened had an AAA, although this value varied from 0.66% to 1.77% depending on geographical location (PHE 2019). This figure represents a continued yearly decline in AAA incidence in England and is the first time it has fallen below 1% (PHE 2019). Similarly, the number of deaths due to AAA rupture has been decreasing over time (Anjum et al 2012). These trends are largely attributable to an overall reduction in the rate of smoking and increases in elective AAA repair in those aged 75 years and over (Anjum et al 2012).

While elective AAA repair is generally associated with low mortality rates, ruptured AAAs (rAAAs) are still associated with an average in-hospital mortality of 35.4% after surgery (National Vascular Registry 2019). In fact, a large-scale systematic review reported that the mortality rate from AAAs reaches a staggering 81% when pre-hospital deaths are included (Reimerink et al 2013).

AAAs also represent a significant economic burden on healthcare systems. In the UK, the estimated hospital cost for the surgical repair of an AAA has been reported as £12,806 for elective cases, and £19,984 for emergency open repair (Thompson et al 2013). Moreover, in 2019, patients admitted with a rAAA in the UK spent a median of eight days in the hospital (NHS 2019). Significant costs are also incurred by national screening programmes, although AAA screening in England has proven highly cost-effective in terms of quality-adjusted life-years gained (Glover et al 2014).

Due to the catastrophic nature of AAA rupture, the National Institute for Health and Care Excellence (NICE), the European Society for Vascular Surgery (ESVS) and the Society for Vascular Surgery (SVS) have recently published comprehensive guidelines on the diagnosis, surveillance and management of AAAs (Chaikof et al 2018, NICE 2020, Wanhainen et al 2019). This review explores these recommendations, highlighting the key preoperative factors that need to be considered to optimise the care that these patients receive.

## Risk factors

### AAA development

Age, gender, ethnicity and prior cardiovascular disease (CVD) are large non-modifiable risk factors for the development of AAAs. After the age of 65 years, the risk of developing an AAA increases by 40% every five years (Vardulaki et al 2000). In addition, a screening study of 2.3 million individuals identified a 6-fold increase in the age-adjusted prevalence of AAAs in men, compared to women (Carter et al 2020). White-British individuals are also at a greater risk of AAA development than black/black-British and Asian/Asian-British individuals (Benson et al 2016), as are those with prior history of CVD. It has recently been shown that, in a cohort with no known CVD, the incidence of AAA is as low as 0.6% (Carter et al 2020). Similarly, patients with other conditions such as connective tissue disorders are at an increased risk of AAA development (Rahimi 2019).

There are various modifiable risk factors that physicians need to consider and communicate to their patients. These include high systolic blood pressure, BMI, serum triglycerides and low-density lipoprotein levels (Summers et al 2020). Perhaps the largest modifiable-risk factor is current smoking. This affects females more than males, with a large cross-sectional study reporting a 15-fold increase in the risk of AAA amongst women compared to a 7-fold increase amongst men (Carter et al 2020). The effects of smoking are also dose-dependent, with a relative risk of 1.78 per every 10 pack-years (Aune et al 2018). Conversely, it has been shown that a linear inverse association exists between the duration of smoking cessation and the risk of AAAs. After 25 years of cessation, this risk approaches that of never-smokers (Aune et al 2018). Other protective factors against AAA development include a healthy diet and exercise more than once a week (Kent et al 2010).

### AAA growth and rupture

AAAs grow insidiously in the absence of symptoms. Each 0.5 cm increase in AAA diameter increases growth rates by 0.5 mm/year and doubles rupture rates (Thompson et al 2013). In smokers, the growth rate is increased by a further 0.35 mm/year and the rupture rate doubles (Thompson et al 2013). The rate of rupture also increases with blood pressure and is almost 4-fold higher in women than men, despite AAAs being more common in males (Thompson et al 2013). Interestingly, diabetes mellitus (DM) may protect against both the expansion and rupture of AAAs, as well as re-growth and the need for re-intervention after endovascular AAA repair (Raffort et al 2018). However, reports also suggest that DM increases intraoperative mortality and the rate of postoperative complications like myocardial infarction, infection and pancreatitis (Raffort et al 2018).

## Screening

A number of countries, including the United Kingdom (UK), have dedicated AAA screening programmes. In the UK, every male is invited for screening within the year of their 65th birthday (PHE 2019). Self-referral to the screening programme is accepted for men over 65 who have not been previously screened, and should be encouraged if any of the following risk factors are present: COPD, coronary, cerebrovascular or peripheral arterial disease, hypertension, hyperlipidaemia, a current or past smoking history and a family history of AAA (NICE 2020, PHE 2019).

**Figure 1.**
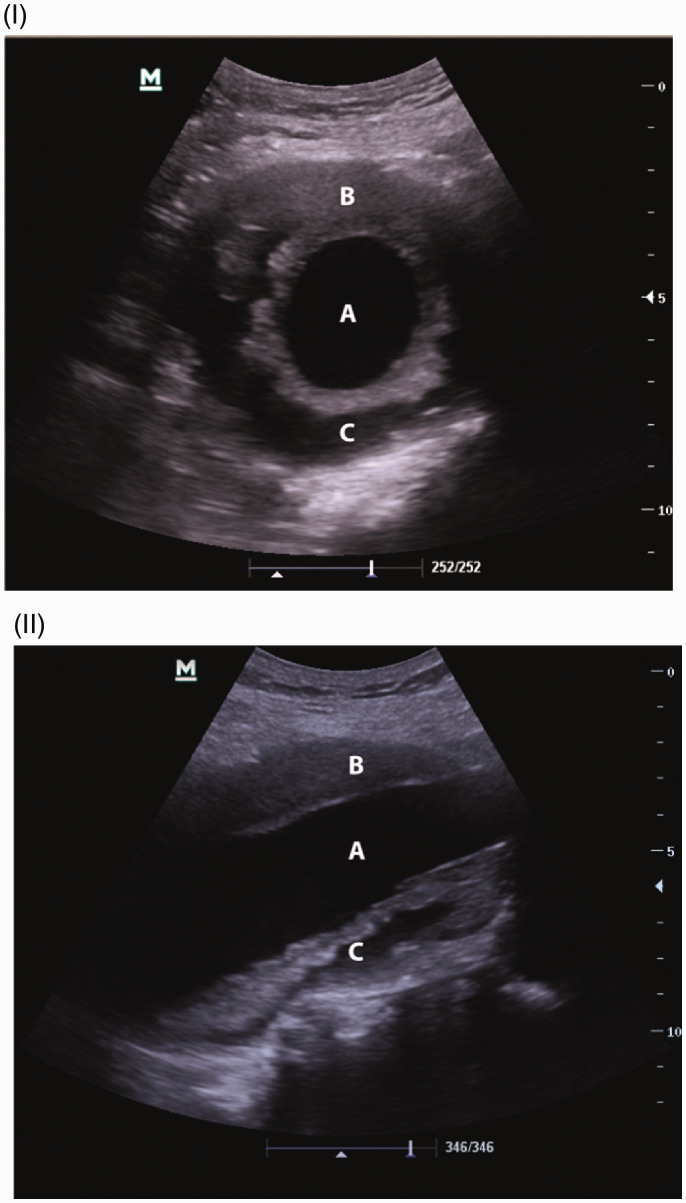
Appearance on ultrasound. (I) Transverse view of an AAA. (II) Longitudinal view. In both, a central lumen (a), an area of stable thrombus (b), and areas of dissection or haemorrhage within the thrombus (c) can be seen. Reproduced with permission from Goldstein and Wells (2019).

Screening involves a single supine abdominal ultrasound scan. The aorta is assessed from the proximal extent of the suprarenal abdominal aorta to the level of the aortic bifurcation and two measurements are taken, one with the probe in the longitudinal plane and the other in the transverse plane (Figure 1; PHE 2016). Specifically, an inner-to-inner maximum anterior-posterior aortic diameter should be taken (NICE 2020). If the abdominal aortic diameter is <3.0cm, it is classed as normal and no further follow-up is required (NHS 2017). An aortic diameter of 3.0–4.4cm is classed as a small AAA, while medium AAAs are between 4.5 and 5.4cm and large AAAs measure ≥5.5cm (NHS 2017). These results are found in approximately 1%, 0.5% and 0.1% of patients, respectively (NHS 2017). Small-to-medium asymptomatic aneurysms are managed conservatively with periodic ultrasound surveillance. This occurs yearly for small AAAs and three monthly for medium AAAs (PHE 2019). Large aneurysms are referred directly to vascular services for diagnosis and treatment (PHE 2019). The indications for surgical repair are discussed later in this article.

Upon diagnosis, patients should be given information regarding AAAs, the risks of growth and rupture, and the treatment options available (NICE 2020). As it may be an emotionally challenging diagnosis to receive, patients should be reassured that most AAAs are unproblematic (NICE 2020). In fact, a UK study of over 5000 men with small or medium AAAs found that mental quality of life was transiently reduced in the year following AAA diagnosis (Bath et al 2018). It is therefore beneficial to offer patients advice and psychological support, for example by referral to a Vascular Nurse Specialist (Rankin 2016).

Between April 2018 and March 2019, 18.7% of eligible men did not undergo screening in the UK (PHE 2019). Reasons for this may include accidental missed appointments, logistical errors, an inability to travel to the screening site and preference. This finding was echoed by a study of national cohorts for two consecutive years, covering 593,032 invitees for screening. Men living in the most deprived decile were significantly more likely to decline screening or not attend their appointment, despite AAAs being twice as common in this population (Jacomelli et al 2017).

Screening for AAA has been shown to significantly reduce mortality rates in 65 to 74-year-old men (Thompson et al 2012). Currently, the national screening programme in the UK does not include females, due to evidence that AAA screening is unlikely to be cost-effective in women (Sweeting et al 2018). This is echoed in the ESVS guidelines, in which cost-effectiveness and harm-benefit analyses are performed (Wanhainen et al 2019). It is, however, acknowledged by NICE and the literature that AAAs are more likely to rupture in women (NICE 2020, Sweeting et al 2012). In fact, women account for one-third of all deaths from a rAAA (Office for National Statistics 2018), despite the prevalence of AAA being approximately six times lower in women (NICE 2020). A recent study in the UK demonstrated that women have a higher in-hospital mortality rate than men after elective AAA repair, irrespective of repair method (Sidloff et al 2017). Therefore, clinicians should retain a high index of suspicion if female patients present with signs and symptoms of an AAA.

### Presentation

The majority of AAAs are asymptomatic and are detected incidentally while investigating a different pathology (Aggarwal et al 2011). In a large study of 79,121 abdominal ultrasound, computed tomography (CT) and magnetic resonance imaging (MRI) scans, an incidental AAA was found in 1% of patients (Van Walraven et al 2010). When symptomatic, an unruptured AAA may present with a pulsating pain or sensation in the stomach or groin (Petriceks et al 2018) and/or a chronic lower back pain from vertebral column erosion (Yildizgoren et al 2016).

As many as 50% of patients with an AAA will have a rAAA as their primary presentation (Jeanmonod et al 2020). This may occur spontaneously or as a result of trauma (Chiriano et al 2010). The classic triad of presentation for a rAAA consists of hypotension, flank or back pain and a pulsatile abdominal mass, typically in a patient over the age of 50 (Yien et al 2008). However, in as many as 50% of patients, this triad may be incomplete (Yien et al 2008). A rAAA should therefore be suspected in any patient >50 years with abdominal pain (RCEM 2019). Other common presentations include shortness of breath, tachycardia and flank or periumbilical ecchymosis (Grey Turner or Cullen signs, respectively) (NHS 2017, Su et al 2020). It is important that doctors are aware of these features to aid their clinical decision-making, as in a six-year retrospective cohort study, 33% of patients with a rAAA were misdiagnosed upon primary assessment, with a trend to more frequent misdiagnosis in women (Smidfelt et al 2017). The most common rAAA misdiagnosis is renal colic (Marston et al 1992).

### Examination

Examination of a suspected AAA should involve using two hands to deeply palpate above and to the left of the umbilicus. Usually, a pulsatile aorta can be felt; however, in cases of AAA, an expansile mass may also be appreciated (Aggarwal et al 2011, Smith-Burgess 2017). Patients with pain and tenderness on abdominal palpation have an increased risk of AAA rupture (Aggarwal et al 2011), but palpation itself is not a precipitating event for rupture (Chaikof et al 2018). It should therefore not deter clinicians from performing a thorough examination. It is also important to auscultate the aorta to detect an aortic bruit, which can be suggestive of arterial atherosclerotic disease (Aggarwal et al 2011). In addition, the femoral and popliteal arteries should be assessed (Chaikof et al 2018). As many as 14% of men with an AAA have been shown to have a coexisting femoral or popliteal aneurysm (Diwan et al 2000).

Although physical examination can be very useful, it only has a moderate sensitivity for detecting AAAs (Aggarwal et al 2011). Moreover, its accuracy is significantly reduced in smaller aneurysms and in patients with a larger BMI (Venkatasubramaniam et al 2004). It is, therefore, important to order the following investigations.

### Investigations

Abdominal ultrasonography is the investigation of choice for suspected AAAs, as well as routine screening (NICE 2020). If this scan detects an aneurysm that measures 3.0–5.4 cm, the patient must be seen at a regional vascular service within 12 weeks (NICE 2020). If the AAA is ≥5.5 cm, the patient must instead be seen within two weeks (NICE 2020). In the emergency setting, bedside ultrasound is also a quick, convenient and reliable imaging modality, with a sensitivity of 96.3% and specificity of 100% for rAAAs (Dent et al 2007). Basic observations and an ECG should also be taken at the bedside, to assess for cardiovascular collapse and peri-arrest rhythms, respectively.

Both unruptured and rAAAs must also be investigated using blood tests. These include a full blood count, urea and electrolytes, liver function tests and blood group (Rahimi 2019). Furthermore, it is important that prior to surgical repair (elective or emergency), the patient is crossmatched as massive blood transfusion is required in 71% of rAAAs (Montan et al 2016).

In addition, NICE recommend that patients undergo thin-slice contrast-enhanced arterial-phase CT angiography (NICE 2020). This allows for anatomical mapping of the AAA in relation to the renal and iliac arteries, planning the appropriate surgical intervention (Boyle et al 2005) and predicting prognosis, for example patients with shorter aneurysm necks have poorer outcomes (IMPROVE 2015). In cases of rAAAs, it can also assess if the rupture is impending or complete, and the extent of aortic wall involvement (Vu et al 2014). However, it is inappropriate to CT unstable, hypotensive patients who need immediate transfer to a vascular surgery centre (NICE 2020). There are no investigations classed as essential for transfer, in order to avoid delay in surgical repair (RCEM 2019).

### Decision for surgical repair

For unruptured AAAs, NICE recommend that clinicians consider surgical repair if patients are either symptomatic, asymptomatic with an AAA >4 cm that has grown >1 cm in one year, or asymptomatic with an AAA ≥5.5cm (NICE 2020). The ESVS guidelines also recommend surgical repair for male patients with an AAA diameter ≥5.5 cm, though the threshold is lowered to ≥5.0 cm for female patients (Wanhainen et al 2019). This is in line with the SVS guidelines, which recommend that for women, surgical repair can be performed between 5 and 5.4cm (Chaikof et al 2018).

The decision for surgical repair must also involve an evaluation of the patient’s age, life expectancy, fitness for surgery and medical comorbidities (NICE 2020). Non-invasive stress testing is recommended for patients with cardiovascular risk factors (Chaikof et al 2018, NICE 2020). An ECG should be performed for all patients within 30-days of a planned intervention and should an active condition be found, referral for cardiac work-up and optimisation is recommended (Chaikof et al 2018, Wanhainen et al 2019). Furthermore, preoperative pulmonary function testing and optimisation, including arterial blood gas analysis, are recommended for patients with risk factors for pulmonary complications or a recent decline in respiratory function (Chaikof et al 2018, Wanhainen et al 2019). Abnormal results may require the administration of bronchodilators for at least two weeks before repair (Chaikof et al 2018). Assessment of renal function should also be conducted for all patients preoperatively. Patients with severe impairment should be referred to a renal physician and will need adequate hydration and monitoring perioperatively (Wanhainen et al 2019). NICE does not advocate the use of risk assessment tools to determine suitability for surgery (NICE 2020).

If surgical repair is deemed unsuitable, the reasoning for this must be clearly and sensitively explained to the patient based on their individual circumstances, alongside alternative management options (NICE 2020). For example, where the AAA is too small to warrant surgery, patients should be informed that elective repair of small or medium aneurysms would offer no long-term survival benefits (Powell et al 2007). Where the patient’s physical condition and comorbidities contraindicate surgery, it should be explained that elective repair poses more risks than benefits for people with poor overall health (NICE 2020).

In comparison, rAAAs are a true surgical emergency and require urgent surgical treatment (Chaikof et al 2018). Patients must be assessed on an individual basis for surgical eligibility and those deemed unlikely to survive may require palliative management instead. Both the NICE and ESVS guidelines emphasise that neither a risk assessment tool nor a single symptom, sign or risk factor should be used to determine suitability for surgery (NICE 2020, Wanhainen et al 2019). This difficult decision must therefore be made based on clinical judgment and expertise.

### Preoperative management

Before elective AAA repair, management concentrates on reducing the risk of rupture (NICE 2020). This involves providing all patients with information, support and interventions for secondary prevention of CVD, including nutritional, exercise and weight-loss advice, diabetes management and anti-platelet therapy (NICE 2020, Wanhainen et al 2019). Patients should be reassured that moderate physical activity is well tolerated and does not precipitate aneurysm growth or rupture (Myers et al 2014). Optimisation may also include blood pressure control and the management of hypercholesterolaemia with statins, which has been shown to improve five-year survival rates in patients with an AAA (Bahia et al 2016, Sweeting et al 2012).

The most impactful intervention is smoking cessation and patients should be referred to a ‘stop smoking service’ where appropriate (Chaikof et al 2018, NICE 2020). Smoking cessation also improves operative outcomes, with ≥8 weeks cessation resulting in a significantly lower likelihood of pulmonary complications, compared to shorter term or a lack of cessation (Arinze et al 2019). Recently, research in animal models has also identified smoking alternatives, namely Electronic Cigarette vapour, as a risk factor for AAA development (Mulorz et al 2019).

Preoperative optimisation of the patient’s medications is also recommended, in accordance with NICE guidelines (NICE 2020). Beta-blockers should be continued throughout the perioperative period but should not be started directly before the surgery, whereas angiotensin-converting enzyme inhibitors should be stopped on the morning of surgery and restarted once euvolaemia is achieved (Chaikof et al 2018). There are currently no pharmacological interventions recommended to prevent AAA growth or rupture (NICE 2020). The European Society for Vascular Surgery has noted that while several classes of drugs have been investigated in randomised trials for their ability to reduce the rate of AAA growth, none have been shown to be effective (Wanhainen et al 2019). However, a clear precipitating cause of a symptomatic AAA, such as an infection, should be treated accordingly, as infection increases rupture risk (Kim 2010). Regardless of the cause, the SVS guidelines recommend antibiotic prophylaxis within 30 min of surgery in the form of an intravenous first-generation cephalosporin or, if penicillin allergic, vancomycin (Chaikof et al 2018).

RAAAs require an urgent ABCDE assessment, stabilisation, analgesia, investigations and preparation for surgery. The regional vascular service should be notified of the rAAA and the required units of cross-matched blood ordered from the blood bank, in accordance with the facilities maximum surgical blood order schedule. The patient should be transferred to a regional vascular service within 30 minutes of the decision to transfer with a restrictive approach to volume resuscitation utilised (NICE 2020).

## Conclusion

AAAs are associated with significant morbidity and mortality. At-risk groups should therefore undergo routine screening and upon diagnosis, receive lifestyle counselling, psychological support and regular monitoring. It is also important to be aware of how symptomatic and rAAAs present, are investigated and are managed in the preoperative period. Depending on the size and/or rupture status of the AAA, patients may then progress to definitive surgical repair.

## Key phrases

1. There are many modifiable and non-modifiable risk factors for AAA development.
2. Ultrasonography is an effective method of screening and monitoring AAAs.
3. AAAs vary in presentation according to their size and rupture status.
4. The preoperative management of stable AAAs concentrates on reducing the risk of rupture.
